# Effect of severe compared with moderate energy restriction on physical activity among postmenopausal female adults with obesity: a prespecified secondary analysis of the Type of Energy Manipulation for Promoting optimum metabolic health and body composition in Obesity (TEMPO) Diet randomized controlled Trial

**DOI:** 10.1093/ajcn/nqac024

**Published:** 2022-01-31

**Authors:** Xingzhong Jin, Alice A Gibson, Zubeyir Salis, Radhika V Seimon, Claudia Harper, Tania P Markovic, Nuala M Byrne, Shelley E Keating, Emmanuel Stamatakis, Elif Inan-Eroglu, Felipe Q da Luz, Julie Ayre, Amanda Sainsbury

**Affiliations:** Centre for Big Data Research in Health, University of New South Wales, Sydney, New South Wales, Australia; The Boden Initiative, Charles Perkins Centre, Faculty of Medicine and Health, The University of Sydney, Sydney, New South Wales, Australia; Sydney Musculoskeletal Health, Kolling Institute, The University of Sydney, Sydney, New South Wales, Australia; The Boden Initiative, Charles Perkins Centre, Faculty of Medicine and Health, The University of Sydney, Sydney, New South Wales, Australia; Menzies Centre for Health Policy and Economics, Faculty of Medicine and Health, The University of Sydney, Sydney, New South Wales, Australia; Centre for Big Data Research in Health, University of New South Wales, Sydney, New South Wales, Australia; The Boden Initiative, Charles Perkins Centre, Faculty of Medicine and Health, The University of Sydney, Sydney, New South Wales, Australia; The Boden Initiative, Charles Perkins Centre, Faculty of Medicine and Health, The University of Sydney, Sydney, New South Wales, Australia; The Boden Initiative, Charles Perkins Centre, Faculty of Medicine and Health, The University of Sydney, Sydney, New South Wales, Australia; Metabolism & Obesity Service, Royal Prince Alfred Hospital, Sydney, New South Wales, Australia; School of Health Sciences, University of Tasmania, Hobart, Tasmania, Australia; School of Human Movement and Nutrition Sciences, The University of Queensland, Brisbane, Queensland, Australia; Charles Perkins Centre, School of Health Sciences, Faculty of Medicine and Health, The University of Sydney, Sydney, New South Wales, Australia; The Boden Initiative, Charles Perkins Centre, Faculty of Medicine and Health, The University of Sydney, Sydney, New South Wales, Australia; Charles Perkins Centre, School of Health Sciences, Faculty of Medicine and Health, The University of Sydney, Sydney, New South Wales, Australia; The Boden Initiative, Charles Perkins Centre, Faculty of Medicine and Health, The University of Sydney, Sydney, New South Wales, Australia; Eating Disorders Program (AMBULIM), Institute of Psychiatry, Faculty of Medicine, University of São Paulo, São Paulo, Brazil; Charles Perkins Centre, School of Health Sciences, Faculty of Medicine and Health, The University of Sydney, Sydney, New South Wales, Australia; School of Human Sciences, The University of Western Australia, Perth, Western Australia, Australia

**Keywords:** obesity, exercise, postmenopause, randomized controlled trial, diet therapy, diet, reducing, very low energy diet, very low calorie diet, sedentary behavior

## Abstract

**Background:**

An under-explored strategy for increasing physical activity is the dietary treatment of obesity, but empirical evidence is lacking.

**Objectives:**

We aimed to compare the effects of weight loss via severe as opposed to moderate energy restriction on physical activity over 36 mo.

**Methods:**

A total of 101 postmenopausal female adults (45–65 y, BMI 30–40 kg/m^2^, <180 min/wk of structured exercise) were randomly assigned to either 12 mo of moderate energy restriction (25%–35% of energy requirement) with a food-based diet, or a severe intervention involving 4 mo of severe energy restriction (65%–75% of energy requirement) with a total meal replacement diet, followed by 8 mo of moderate energy restriction. Physical activity was encouraged, but no tailored or supervised exercise prescription was provided. Physical activity was assessed with an accelerometer worn for 7 d before baseline (0 mo) and 0.25, 1, 4, 6, 12, 24, and 36 mo after intervention commencement.

**Results:**

Compared with the moderate group, the severe group exhibited greater mean: total volume of physical activity; duration of moderate-to-vigorous-intensity physical activity (MVPA); duration of light-intensity physical activity; step counts, as well as lower mean duration of sedentary time. All these differences (except step counts) were apparent at 6 mo [e.g., 1006 metabolic equivalent of task (MET)-min/wk; 95% CI: 564, 1449 MET-min/wk for total volume of physical activity], and some were also apparent at 4 and/or 12 mo. There were no differences between groups in the 2 other outcomes investigated (self-efficacy to regulate exercise; and proportion of participants meeting the WHO's 2020 Physical Activity Guidelines for MVPA). When the analyses were adjusted for weight at each time point, the differences between groups were either attenuated or abolished.

**Conclusions:**

Among female adults with obesity, including a dietary component to reduce excess body weight—notably one involving severe energy restriction—could potentially enhance the effectiveness of physical activity interventions.

This trial was registered at www.anzctr.org.au as ACTRN12612000651886.

## Introduction

Physical inactivity is a modifiable risk factor for a range of chronic conditions, including hypertension, type 2 diabetes, cardiovascular disease, cancer, osteoarthritis, depression, and dementia (1), and a key contributor to the obesity epidemic (2). The WHO's Global Action Plan on Physical Activity 2018–2030 has set a target of reducing physical inactivity by 10% by the year 2025 and by 15% by the year 2030 (3). However, the prevalence of physical inactivity in high-income countries increased by 13.6% between 2001 and 2016 (from 31.5% to 35.8%) (4). These data highlight the challenges of meeting the WHO's target by 2030, and the urgent need for effective strategies to increase physical activity.

A potential but under-explored strategy for increasing physical activity is the dietary treatment of obesity (i.e., restriction of energy intake for weight loss). Physical inactivity and obesity are bidirectionally related, in that physical inactivity contributes to a positive energy balance, and obesity contributes to physical inactivity (5, 6). As examples of the latter, people with obesity experience physical and psychosocial barriers to physical activity (7), such as pain, impaired mobility, stigmatization, and lack of confidence. These barriers to physical activity may be reduced by dietary obesity treatments, because energy restriction and the resultant weight loss reduce pain (8), increase mobility (9), and enhance self-esteem and body image (10). Thus, weight loss may help to increase physical activity in people with obesity. However, research to date has focused on how physical activity affects weight loss, as opposed to how weight loss affects physical activity. Consequently, it is unclear whether—and to what extent—physical activity changes in response to dietary treatments of obesity.

Although it may be plausible that dietary treatments of obesity could increase physical activity, they could also conceivably decrease physical activity. Indeed, a reduction in physical activity—and/or the energy cost of physical activity—has been observed during energy restriction with weight loss in humans and other species, as previously reviewed (11, 12). Some evidence suggests that these effects may dissipate after restrictions on energy intake are removed (13) (i.e., during weight maintenance after weight loss), but this has never been systematically investigated. Further, different approaches to the dietary treatment of obesity may have different effects on physical activity, in both the short term and long term. For instance, compared with traditional food-based moderately energy-restricted diets, severely energy-restricted diets, a broad term used to describe very-low-energy diets (<3300 kJ/d or <800 kcal/d) and low-energy diets (<5000 kJ/d or <1200 kcal/d), which are typically administered using meal replacement products, may induce feelings of lethargy or light-headedness (14), which could potentially hinder physical activity. Alternatively, severely energy-restricted diets could potentially increase physical activity during adherence to the diet, via mildly elevated blood concentrations of endogenously produced ketone bodies (15), which are associated with a state of euphoria and which could potentially promote physical activity (16). Indeed, some clinical trials showed that supplementation with exogenously administered ketone bodies enhanced certain measures of physical performance, albeit a recent systematic review found equivocal and inconclusive findings (17). However, any such effect of endogenously produced ketone bodies, if present, would likely dissipate upon reintroduction of food and weight stabilization.

Taken together, there are theoretical reasons to assume that different dietary treatments of obesity could increase or decrease physical activity in the short term and long term, during and after energy restriction, but empirical evidence is lacking. To this end, we compared the effects of weight loss via severe as opposed to moderate energy restriction for the treatment of obesity on physical activity over sequential time points up to 36 mo (156 wk).

## Methods

This article reports on a secondary analysis of the TEMPO (Type of Energy Manipulation for Promoting optimum metabolic health and body composition in Obesity) Diet Trial (ANZCTR ACTRN12612000651886). This secondary analysis is reported in line with the Consolidated Standards of Reporting Trials (CONSORT) statement. The TEMPO Diet Trial was a single-center randomized controlled trial that aimed to assess the long-term effects of severe compared with moderate energy restriction on body composition and cardiometabolic health in postmenopausal female adults with obesity. The trial was conducted at the Charles Perkins Centre Royal Prince Alfred Clinic on the University of Sydney campus in Sydney, New South Wales, Australia. Ethical approval was obtained from the Sydney Local Health District Ethics Review Committee, Royal Prince Alfred Hospital Zone. Written informed consent was obtained from all participants before participation in this study. The detailed protocol, including all eligibility criteria, for the TEMPO Diet Trial has been published previously (18), with salient points outlined in what follows. Key inclusion criteria were female adults aged 45–65 y with BMI (in kg/m^2^) between 30 and 40, ≥5 y after menopause (i.e., ≥5 y after the last period of menstrual bleeding, as assessed by answering a screening question that was asked on 3 separate occasions—once via email, once over the phone, and once face-to-face, in-person), sedentary [defined as <180 min (3 h) per week of structured exercise], and living in the Sydney metropolitan area of New South Wales, Australia.

### Randomization and masking

Participants were randomly assigned using stratified permuted blocks. Participants were stratified by BMI (<35 and ≥35) and age (<55 y and ≥55 y), and the 4 stratified groups were randomly assigned in blocks of 2 with a 1:1 ratio into severe and moderate energy restriction intervention groups. Randomization was undertaken by an investigator who was not involved in data collection or intervention delivery. Neither the researchers who collected the data nor the participants themselves were masked to intervention group allocation, because the 2 dietary interventions were clearly discernible (e.g., from the use or not of meal replacement products, and from the rate of weight loss). However, data were objectively collected using an accelerometer and an online questionnaire, and the data were read by the associated software (details follow) to avoid measurement bias.

### Interventions

Full details of the interventions used in this trial have been published previously (19); a summary of points pertinent to this study follows.

#### Moderate intervention

The moderate intervention entailed a 12-mo food-based diet with an energy intake prescription designed to achieve an energy restriction of 25%–35% relative to estimated energy expenditure. The food-based diet was based on the Australian Guide to Healthy Eating (20). It was designed to meet the nutritional requirements of female adults of the age group recruited to this trial, while still being within our target for energy intake.

#### Severe intervention

The severe intervention used a commercially available total meal replacement diet (KicStart^TM^ meal replacement products, Prima Health Solutions Pty Ltd) for 4 mo (16 wk) or until reaching a BMI of 20, whichever came first, followed by the moderate intervention for the remaining time up until 12 mo. The total meal replacement diet was designed to achieve an energy restriction of 65%–75% relative to the estimated energy expenditure of each participant.

#### Prescription for physical activity

Participants in both intervention groups were given a consumer-facing pedometer (Omron HJ-203) with which to monitor their step counts, and brief verbal advice to aim for a total of 8000–12,000 steps per day and to gradually build up to doing 30–60 min of moderate-to-vigorous-intensity physical activity (MVPA) per day, as per our published protocol (18). No tailored or supervised physical activity intervention or advice was provided in this trial.

#### Clinical support

As previously described (18) and directly quoted here: "participants were required to attend 21-22 individual dietary appointments with the trial dietitian (AAG or CH). The initial individual dietary appointment at week 0 was scheduled for approximately 90 min, which is also when participants in the [severe] intervention received their first meal replacement products (shakes and soups) and protein supplementation as required [(19)]. Subsequent individual dietary appointments (for review) were scheduled for 30 min approximately every 2 weeks for the first 26 weeks of the intervention (i.e., at 1, 2, 4, 6, 8, 10, 12, 15, 16, 18, 20, 25, and 26 weeks relative to commencement of the dietary interventions, plus an extra appointment at 17 weeks for participants in the [severe] intervention during their transition from the [severe] to the [moderate] intervention), and then approximately every month until 52 weeks (i.e., at 29, 33, 37, 41, 45, 51, and 52 weeks). To increase compliance with individual dietary appointments, participants were able to complete appointments that did not require face-to-face contact (i.e., to collect a food, activity, and sleep diary or to collect meal replacement products and protein) via telephone. After 52 weeks, participants were given the option of attending monthly group support meetings of 60-90 min in duration each, facilitated on a rotating basis by different members of the research team (AAG, RVS, CH, FQdL and AS), sometimes in association with a guest facilitator."

### Dietary adherence measures

As we have previously stated (21) and as directly quoted here: "since the use of food diaries to measure adherence to the prescribed diet is difficult to assess because of missing dietary records and underreporting among participants with overweight and obesity [(22)], weight loss was used to monitor adherence to the diets [(23)]. We expected approximately 1.5 to 2.5 kg/wk weight loss for participants in the severe intervention [(24)] and approximately 0.5 to 1.0 kg/wk weight loss for participants in the moderate intervention [(25)]."

### Outcome measures

#### Accelerometry

Physical activity was assessed using an accelerometer (SenseWear Pro Armband, BodyMedia Inc.) and was recorded at 1-min intervals. This accelerometer has previously been validated for the measurement of physical activity in older adults in free-living conditions (26). Participants were asked to wear the accelerometer on the back of the upper left arm, over the triceps muscle, for 7 consecutive days before each visit to our clinical research facility for data collection, which for this study occurred at months 0 (baseline), 0.25, 1, 4, 6, 12, 24, and 36. If the participant experienced skin irritation from the accelerometer, they were encouraged to wear the accelerometer on alternate arms on alternate days. The time points of 24 and 36 mo are in addition to the time points listed in our prospective clinical trial registration (i.e., 0, 0.25, 1, 4, 6, and 12 mo). The reason for this discrepancy is that after this trial was registered in 2012, an article was published (in 2014) showing that the effects of an energy-restricted diet on appetite persisted when measured at 24 and 36 mo after diet commencement (27). We therefore hypothesized that any effects of our energy-restricted diets on physical activity may also persist when measured at 24 and 36 mo after diet commencement, hence these additional time points.

Physical activity data were used if participants wore the accelerometer for a minimum of 2 weekdays and 1 weekend day of the 7 d for ≥21 h (87.5%) of each of those days. Participants were also asked to record any times and reasons for taking off the accelerometer in a food, activity, and sleep diary that they were asked to keep for each of those 7-d measurement periods. For example, if they took off the accelerometer for showering or swimming (because the accelerometer was not waterproof), they were asked to record that in their diary. Data were analyzed using the SenseWear software version 8.1 according to the manufacturer's manual.

#### Main outcome of interest

All outcomes reported in the current article are secondary outcomes from a larger trial (the TEMPO Diet Trial). The main outcome of interest to the current secondary analysis was the total weekly volume of physical activity [defined as any activity with an intensity ≥1.5 metabolic equivalents of task (METs)], expressed in MET-min (the intensity of the activity in METs multiplied by the number of minutes at that intensity), at each time point during the trial. For example, a person who was active at an intensity level of exactly 1.5 METs for 100 min in a week (or exactly 3.0 METs for 50 min) undertook 150 MET-min of physical activity that week. This physical activity outcome was of greatest relevance to the current secondary analysis because the total volume of physical activity is likely to be more important for weight management than any other aspect of physical activity (28).

#### Additional outcomes

Additional outcomes for this secondary analysis were weekly duration of physical activity at different intensity levels (light, 1.5 to <3.0 METs; moderate, 3.0 to <6.0 METs; vigorous, 6.0 to <9.0 METs; and very vigorous, ≥9.0 METs). We decided a priori that if there were insufficient instances of vigorous/very vigorous physical activity in the data, moderate and vigorous/very vigorous physical activity would be combined and reported as duration per week of MVPA (≥3.0 METs). Other additional outcomes for this secondary analysis were daily step counts (recorded with the SenseWear Pro Armband); daily sedentary time (i.e., activities at <1.5 METs during waking hours, which would include sedentary activities such as sitting or lounging, and stationary activities such as standing still); and score on a self-efficacy to regulate exercise (SEREx) scale (29). The SEREx scale assessed participants’ confidence in their ability to exercise regularly (≥3 times/wk) in 18 situations which are sometimes reported to jeopardize adherence to an exercise routine, such as after stopping regular exercise owing to illness. Possible scores for each question ranged from 0—“cannot do at all,” to 100—“highly certain can do,” and the overall score was the mean of all 18 questions. The final additional outcome for this secondary analysis was the proportion of participants meeting the higher threshold of the WHO 2020 Physical Activity Guidelines for MVPA, which is to “do at least 150-300 minutes of moderate-intensity aerobic physical activity; or at least 75-150 minutes of vigorous-intensity aerobic physical activity; or an equivalent combination of moderate- and vigorous-intensity activity throughout the week, for substantial health benefits” (30). We decided that the higher threshold (i.e., 300 min of moderate-intensity physical activity or equivalent) was a more appropriate threshold for the current secondary analysis than the lower threshold (i.e., 150 min of moderate-intensity physical activity or equivalent), because 200–300 min/wk of moderate-intensity physical activity is recommended for preventing weight regain after weight loss (28).

### Statistical analysis

Because this article reports secondary outcomes from a larger trial (the TEMPO Diet Trial), no power calculations were performed for any of the outcomes reported in this article. The sample size calculation for the TEMPO Diet Trial was based on detecting a between-group difference of 5% in whole-body lean mass at 12 mo after intervention commencement (21). We calculated that a target sample size of 100 participants would provide a power of 90% at a 2-sided α level of 5%, allowing for ≤20% loss to follow-up.

Statistical comparison of longitudinal changes between groups was performed on an intention-to-treat basis. We used Little's test to determine whether missing data were “missing completely at random.” Comparisons between intervention groups for the 6 continuous outcome variables investigated in this study were assessed using repeated-measures linear mixed-effect models, with missing data handled by the restricted maximum likelihood estimation function in the linear mixed-effect models. Intervention group and time point, as well as the interaction between intervention group and time point, were included as covariates in the repeated-measures linear mixed-effect models as “fixed effects,” as was the baseline value of the outcome under investigation (i.e., the value at month 0), as recommended previously (31). Further justification for our decision to include the baseline value of the outcome variable under investigation as a covariate is the fact that it had a significant influence on the results of the analyses of all continuous variables. The correlation between repeated measures was factored into our repeated-measures linear mixed-effect models by setting individual participant identification as a random intercept (“random effect”). When the overall *P* value for the interaction between intervention group and time point was <0.05, pair-wise comparisons of the estimated marginal means (means after adjusting for covariates) between intervention groups at all of the 7 time points were conducted, using a Bonferroni-adjusted *P* value threshold for statistical significance of 0.0071 (i.e., 0.05 divided by the number of comparisons being made, which was 7).

In addition to assessing between-group differences as outlined already, we also used the aforementioned repeated-measures linear mixed-effect models to assess within-group differences [i.e., values at baseline (month 0) compared with values at all other time points within each intervention group]. We did this because health care providers and consumers may be interested to know the effect of a particular diet compared with not doing any diet at all, in addition to the effect of one diet compared with another. Given that we did not have a no-diet control group (which could be considered unethical for our study population, given the known health benefits of dietary weight loss interventions for people with obesity, as was true of participants in this trial), comparison of baseline (month 0) values with all other time points was conducted, again using a Bonferroni-adjusted *P* value threshold for statistical significance of 0.0071 as described already.

To determine the possible impact of differences in weight between the 2 intervention groups (i.e., to control for the fact that the severe intervention resulted in greater mean weight losses than the moderate intervention), we reran the repeated-measures linear mixed-effect models using weight at each time point as a fixed-effect covariate in the analyses.

Normality of each continuous outcome variable was assessed by visual inspection of *1*) a frequency histogram of each continuous outcome variable; and *2*) a quantile-quantile (Q-Q) plot of the quantile of each data point for each continuous outcome variable, plotted against the value of an equivalent quantile from a normal distribution. Because our data were normally distributed, we did not perform data transformations. The assumptions of the repeated-measures linear mixed-effect models used for our analyses of continuous outcome variables were assessed by visually inspecting 2 plots for each analysis of each continuous outcome variable: *1*) the residuals of each observed (raw) data point from the corresponding “fitted” data point predicted from the model, plotted against the corresponding fitted data point (this plot should show no obvious pattern); and *2*) a Q-Q plot of the quantile of each residual for each observed (raw) data point from the corresponding “fitted” data point predicted from the model, plotted against the value of an equivalent quantile from a normal distribution (which should produce a straight line with a positive slope).

The binary outcome variable of meeting or not meeting the upper threshold of the WHO 2020 Physical Activity Guidelines (30) for MVPA was analyzed with a generalized mixed-effect model with logit function within the binomial family, with missing data handled by the restricted maximum likelihood estimation function. The covariance structure in the aforementioned generalized mixed-effect model was assumed to be unstructured. This generalized mixed-effect model was made up of a “fixed-effect” component—which comprised the covariates of intervention group, time point, and baseline outcome (i.e., meeting or not meeting the guidelines)—and a “random-effect” component, which comprised individual participant identification. Note that the interaction between intervention group and time point was not statistically significant in this generalized mixed-effect model and so was not included as a covariate.

All analyses were performed in R version 4.1.2 (R Foundation). The null hypothesis was tested with a 2-tailed test.

## Results

**FIGURE 1:**
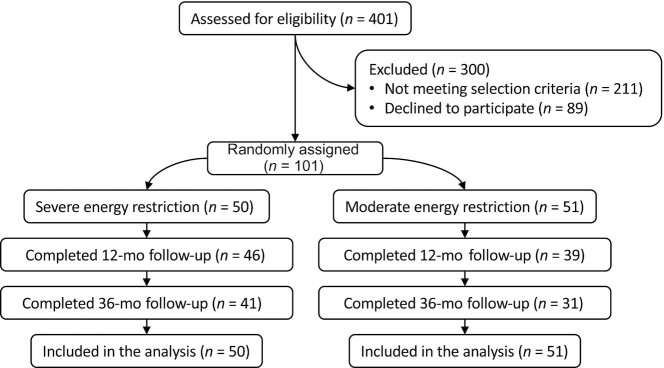
Flow of participants in the TEMPO Diet Trial. TEMPO, Type of Energy Manipulation for Promoting optimum metabolic health and body composition in Obesity.

A total of 101 participants were recruited to the TEMPO Diet Trial between March 2013 and July 2016, and were randomly assigned to either the severe (*n* = 50) or moderate (*n* = 51) intervention (**Figure 1**). We completed recruitment at this time because our target of 100 participants had been met. The mean ± SD age of participants was 57.5 ± 4.2 y, and the mean ± SD BMI was 34.5 ± 2.5. Overall, 85 (84.2% of) participants [46 of 50 (92.0%) in the severe group and 39 of 51 (76.5%) in the moderate group] completed the 12-mo intervention, and 72 (71.3% of) participants [41 of 50 (82.0%) in the severe group and 31 of 51 (60.8%) in the moderate group] completed the 36-mo follow-up (21, 32). The trial ended as planned, when the last follow-up data from the last participant were collected, which was in September 2019. At baseline, participants’ age and anthropometry (i.e., weight, height, and BMI) and the outcome variables addressed in this study appeared comparable between the 2 intervention groups, and there were no apparent differences between participants who completed the study and those who did not (**Table 1**). Mean weight loss during the first 4 mo [published previously (21)] was 44%–73% of that expected for the severe intervention [i.e., mean weight loss was 17.4 kg (21), or 1.1 kg/wk, compared with an expected 1.5–2.5 kg/wk (24)] and was 40%–80% of that expected for the moderate intervention [i.e., mean weight loss was 7.1 kg (21), or 0.4 kg/wk, compared with an expected 0.5–1.0 kg/wk (25)]. These findings suggest 40%–80% compliance with the interventions in the first 4 mo, with lower rates of weight loss after 4 mo (21) suggesting lower adherence.

**TABLE 1:** Baseline characteristics of age, anthropometric measures, physical activity, and related parameters in participants of the TEMPO (Type of Energy Manipulation for Promoting optimum metabolic health and body composition in Obesity) Diet Trial^1^

	Severe energy restriction			Moderate energy restriction		
	All participants (*n* = 50)	Completers (*n* = 41)	Noncompleters (*n* = 9)	All participants (*n* = 51)	Completers (*n* = 31)	Noncompleters (*n* = 20)
Age, y	57.5 ± 4.3	57.7 ± 4.3	56.7 ± 4.2	57.5 ± 4.1	57.2 ± 4.2	58.0 ± 4.1
Weight, kg	90.1 ± 9.4	89.2 ± 8.9	94.5 ± 11.1	92.4 ± 8.3	93.3 ± 9.0	91.0 ± 6.9
Height, cm	161.9 ± 6.1	162.0 ± 5.7	161.0 ± 7.9	163.3 ± 5.3	163.0 ± 6.0	163.0 ± 4.2
Body mass index, kg/m^2^	34.3 ± 2.5	33.9 ± 2.4	36.2 ± 1.8	34.6 ± 2.5	35.0 ± 2.7	34.1 ± 2.2
Total volume of PA, MET-min/wk	3724 ± 1377	3697 ± 1472	3850 ± 865	3627 ± 1412	3781 ± 1384	3376 ± 1457
MVPA, min/wk	319 ± 187	328 ± 195	279 ± 148	327 ± 209	336 ± 224	312 ± 189
Light-intensity PA, min/wk	1340 ± 482	1308 ± 495	1488 ± 411	1275 ± 501	1326 ± 518	1192 ± 474
Steps, count per day	7153 ± 2664	7218 ± 2737	6856 ± 2422	7133 ± 2499	7390 ± 2308	6714 ± 2797
Sedentary time, min/d	1163 ± 93	1168 ± 98	1138 ± 59	1176 ± 87	1172 ± 86	1182 ± 89
Self-Efficacy to Regulate Exercise score (range: 0–100)	43.4 ± 18.9	50.2 ± 24.5	30.8 ± 20.7	46.6 ± 24.8	44.0 ± 19.9	42.5 ± 17.7
Proportion meeting current guidelines for MVPA	24 (48.0)	20 (48.8)	4 (44.4)	23 (45.1)	15 (48.4)	8 (40.0)

1 Values are mean ± SD or *n* (%) of all available data at baseline (month 0). Missing data were not imputed. MET, metabolic equivalent of task; MVPA, moderate-to-vigorous-intensity physical activity; PA, physical activity.

Over 98% of our accelerometry data were usable, in that participants remaining in the trial wore the accelerometer for a minimum of 2 weekdays and 1 weekend day for ≥21 h (87.5%) of each day. Little's test of missing completely at random for accelerometry data (using total weekly volume of physical activity as an example) was nonsignificant (*P* = 0.99), and thus we concluded that the missing accelerometry data were missing at random. Because there were only 7 occasions when a participant recorded removing the accelerometer for water-based physical activity (e.g., swimming, surfing), these manually recorded activities were not included in our estimates of physical activity. We saw very little vigorous activity in our trial participants (<6.5 min/wk on average across all time points), so we merged moderate-intensity and vigorous-intensity physical activity and reported the duration of MVPA (≥3.0 METs).

**FIGURE 2:**
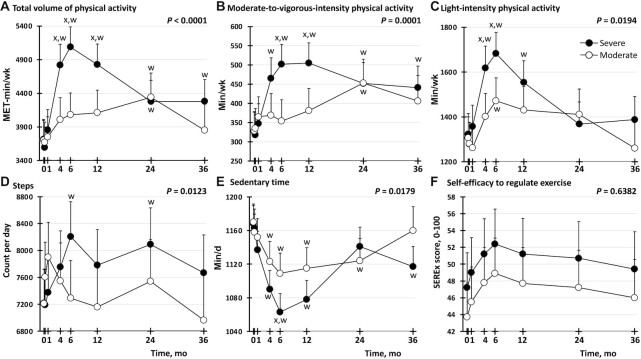
Effect of severe compared with moderate energy restriction on physical activity in postmenopausal female adults with obesity. (A) Total volume of physical activity, (B) moderate-to-vigorous-intensity physical activity, (C) light-intensity physical activity, (D) step count, (E) sedentary time, (F) self-efficacy to regulate exercise. Analyses were performed on an intention-to-treat basis (*n* = 101: 50 in the severe intervention; 51 in the moderate intervention). Data shown are estimated marginal means (i.e., group means after controlling for covariates), with whiskers indicating the upper ranges of 95% CIs from a repeated-measures linear mixed-effect model. Missing data were handled by the restricted maximum likelihood estimation function in the linear mixed-effect model. The *P* value shown at the top right of each panel is for the intervention group × time point interaction for the outcome shown in that panel. ^x^*P* value for the pair-wise comparison across intervention groups at that time point is <0.0071, the Bonferroni-adjusted threshold for statistical significance (i.e., 0.05 divided by the number of comparisons being made, which was 7 because there were 7 time points). ^w^*P* value for the pair-wise comparison from baseline (month 0) within that intervention group to that time point is below the Bonferroni-adjusted threshold of 0.0071. MET, metabolic equivalent of task; SEREx, self-efficacy to regulate exercise.

As shown in **Figure 2**, mean physical activity was greater in the severe group than in the moderate group, and mean sedentary time was lower in the severe than in the moderate group. These between-group differences are apparent in Figure 2 from the *P* values for the interaction between intervention group and time point, which were <0.05 for all of the continuous outcomes of physical activity/sedentary time investigated in this study (i.e., total weekly volume of physical activity; weekly duration of MVPA; weekly duration of light-intensity physical activity; daily step count; and daily duration of sedentary time). Pairwise comparisons between intervention groups at each time point (i.e., at 0, 0.25, 1, 4, 6, 12, 24, and 36 mo) showed *P* values less than our Bonferroni-adjusted threshold of 0.0071 at the 6-mo time point for all these continuous outcomes except daily step count (Figure 2, **Supplemental Table 1**). At this time point (6 mo), and compared with the moderate group, the severe group exhibited 1006 (95% CI: 564, 1449) more MET-minutes per week for total volume of physical activity; 147 (95% CI: 71, 223) more minutes per week of MVPA; 210 (95% CI: 71, 350) more minutes per week of light-intensity physical activity; and 46 (95% CI: 14, 78) fewer minutes per day of sedentary time (Supplemental Table 1). In addition to these between-group differences at 6 mo, there were between-group differences with *P* values <0.0071 at 4 and/or 12 mo for total weekly volume of physical activity, weekly duration of MVPA, and weekly duration of light-intensity physical activity, as shown in Figure 2. For daily step count, although there was an overall difference between intervention groups as aforementioned [as indicated by the *P* value <0.05 for the interaction between intervention group and time point (i.e., *P* = 0.0123); Figure 2], there was no between-group difference in daily step count with a *P* value <0.0071 (Figure 2D), indicating a weak effect. This finding suggests that the greater levels of physical activity observed in the severe group than in the moderate group may have been due to greater engagement in activities such as cycling or resistance training (as opposed to walking), because activities such as cycling or resistance training are detected by the galvanic skin response mechanism of the accelerometer, but not by the pedometer.

When compared with baseline, the severe group exhibited clear within-group increases in physical activity and reductions in sedentary time (Figure 2). This is denoted by the “w” symbols near specific filled data points in Figure 2, as well as by *P* values below the Bonferroni-adjusted threshold of 0.0071 in the second column of Supplemental Table 1. These differences from baseline in the severe group were first apparent at 4 mo after commencement of the intervention (at which time point participants were coming to the end of the 4-mo severely energy-restricted diet), except for step counts, where the difference was first apparent at 6 mo. The within-group differences from baseline in the severe group were observed until ≥12 mo after commencement of the 12-mo intervention (Figure 2). Even at 36 mo, which is 24 mo after completion of the 12-mo intervention, the severe group demonstrated greater total volume of physical activity (Figure 2A) and duration of MVPA (Figure 2B), and lower sedentary time (Figure 2E), than at baseline. In contrast to the severe group, the moderate group exhibited within-group increases from baseline at only 1 time point (i.e., at 24 mo for each of total volume of physical activity and duration of MVPA, and at 6 mo for light-intensity physical activity, as shown by the “w” symbols near these open data points in Figure 2, and by *P* values <0.0071 in the third column of Supplemental Table 1). The moderate group also exhibited within-group reductions from baseline in sedentary time at each of 4, 6, 12, and 24 mo (Figure 2).

There were no significant differences between groups in SEREx score at any time point, and no changes from baseline in either group (Figure 2F).

**FIGURE 3:**
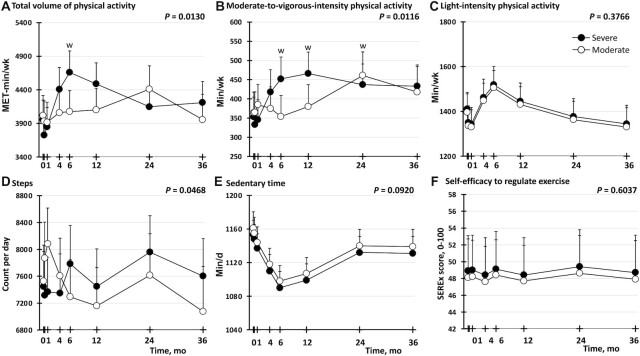
Effect of severe compared with moderate energy restriction on physical activity in postmenopausal female adults with obesity, adjusted for weight. (A) Total volume of physical activity, (B) moderate-to-vigorous-intensity physical activity, (C) light-intensity physical activity, (D) step count, (E) sedentary time, (F) self-efficacy to regulate exercise. Analyses were performed on an intention-to-treat basis (*n* = 101: 50 in the severe intervention; 51 in the moderate intervention). Data shown are estimated marginal means (i.e., group means after controlling for covariates), with whiskers indicating the upper ranges of 95% CIs from a repeated-measures linear mixed-effect model that included weight at each time point as a covariate. Missing data were handled by the restricted maximum likelihood estimation function in the linear mixed-effect model. The *P* value shown at the top right of each panel is for the intervention group × time point interaction for the outcome shown in that panel. ^w^*P* value for the pair-wise comparison from baseline (month 0) within that intervention group to that time point is below the Bonferroni-adjusted threshold of 0.0071 for statistical significance (i.e., 0.05 divided by the number of comparisons being made, which was 7 because there were 7 time points). MET, metabolic equivalent of task; SEREx, self-efficacy to regulate exercise.

We noted that the pattern of differences between groups in physical activity was similar to the previously published (21, 32) pattern of differences between groups in weight. Specifically, differences between groups in physical activity (total weekly volume of physical activity; weekly duration of MVPA; and weekly duration of light-intensity physical activity) and sedentary time were only seen at one or more of 4, 6, and 12 mo (Figure 2), which are the time points when the weight differences between groups were most marked (21, 32). This led us to hypothesize that the differences in physical activity and sedentary time between intervention groups could be mediated by differences in weight between the groups. To assess this exploratory hypothesis statistically, we reran the repeated-measures linear mixed-effect models that were used to generate Figure 2, this time including weight at each time point. As seen in **Figure 3** and **Supplemental Table 2**, when the data were in this way “adjusted for weight,” the differences between intervention groups were either attenuated (for total weekly volume of physical activity; and weekly duration of MVPA) or abolished (for weekly duration of light-intensity physical activity; daily step count; and sedentary time). For the outcomes that were attenuated but not abolished by adjusting for weight (i.e., total weekly volume of physical activity; and weekly duration of MVPA), the *P* values for the interaction between intervention group and time point remained <0.05, but the effect was weak because pairwise comparisons between intervention groups at each time point did not reveal any differences with a *P* value <0.0071 at any specific time point (Figure 3). Not only were the differences between intervention groups attenuated or abolished by adjusting for weight at each time point, the differences within intervention groups from baseline were also attenuated (for total weekly volume of physical activity; and weekly duration of MVPA in the severe group) or abolished (for all other outcomes for the severe group, and for all outcomes for the moderate group) (Figure 3, Supplemental Table 2). Thus, the greater physical activity observed in the severe intervention group than in the moderate intervention group appears to be partially—not completely—related to the fact that the severe intervention group lost more weight than the moderate intervention group.

Despite the differences between intervention groups in duration of MVPA, there was no significant effect of intervention group (*P* = 0.1797) or time point (or the interaction between them) on the proportion of participants meeting the upper threshold of the WHO 2020 Physical Activity Guidelines (30) for recommended weekly duration of MVPA (data not shown).

## Discussion

This analysis of objectively measured physical activity suggests that a 12-mo dietary obesity treatment involving 4 mo consuming a severely energy-restricted total meal replacement diet resulted in greater amounts of physical activity and lower amounts of sedentary time than a 12-mo moderately energy-restricted food-based diet. These differences between dietary obesity treatments (e.g., up to 147 more minutes of MVPA per week) were first apparent at 4–6 mo, and persisted up to 12 mo of the 36-mo randomized controlled trial. The differences occurred without any tailored or supervised exercise prescription. The apparently greater physical activity in the severe than in the moderate dietary treatment was partially but not completely related to the greater weight loss achieved with the severe treatment [e.g., 19.6% compared with 7.7% of initial weight at 4 mo, and 17.3% compared with 8.8% at 12 mo (21)].

In postmenopausal female adults with obesity, which was the participant group in the current trial, weight loss and fat loss have been shown to be more likely or greater when achieved with the combination of dietary energy restriction plus exercise than when achieved with dietary energy restriction alone or exercise alone (33). The findings from the current study would thus imply that for postmenopausal female adults with obesity, severely energy-restricted meal replacement diets would be of benefit, in part by virtue of promoting greater weight loss and greater physical activity. Because severely energy-restricted meal replacement diets are being increasingly used in the treatment of obesity, such as in the publicly funded rollout of these diets for people with obesity and diabetes in the United Kingdom (34), our findings are relevant to the increasing number of people who are using severely energy-restricted meal replacement diets for the management of obesity.

In our Introduction section, we hypothesized—based on literature (15–17)—that the elevated circulating ketone body concentrations observed during a severely energy-restricted diet may contribute to alterations in physical activity. However, this is unlikely because unpublished data from our team show that the differences between dietary intervention groups in circulating ketone body concentrations were only apparent between 0.25 and 4 mo inclusive (i.e., during the time when participants were on the total meal replacement diet; Seimon RV, McClintock S, Salis Z, Inan-Eroglu E, Gibson AA, Harper C, Das A, Roekenes J, King N, Markovic TP, Franklin J, Caterson ID, Byrne NM, Sainsbury A, unpublished results, 2022), whereas the differences between dietary intervention groups in physical activity were apparent at 4, 6, and 12 mo, inclusive. Similarly, it would seem unlikely that the differences in physical activity between the 2 intervention groups at 4, 6, and 12 mo were due to difference between the 2 intervention groups in energy restriction per se, because the difference in energy restriction (prescribed) was only present until 4 mo inclusive—the same time frame in which differences in circulating ketone body concentrations were apparent. After 4 mo, both groups were prescribed the same energy restriction (i.e., the moderate intervention). These observations are in keeping with our finding that part of the effect of the dietary interventions on physical activity (and apparently all of the effect on sedentary time) were mediated by the effects of the interventions to induce weight loss. Possible mechanisms by which weight loss could have promoted physical activity and reduced sedentary time in this trial are the known effects of weight loss to reduce pain (8), increase mobility (9), and enhance self-esteem and body image (10). However, the severe diet had advantages for increasing total weekly volume of physical activity and increasing weekly duration of MVPA over and above its effects to induce greater weight loss than in the moderate group, and the reason for this is unclear from the current analysis.

To our knowledge, this is the first randomized controlled trial to investigate the long-term (3-y) impact of different dietary obesity treatments on objectively measured physical activity. Previous trials and observational studies have focused on the effect of physical activity on weight loss or weight maintenance, as previously reviewed (35). Another strength of this study is that participants were only given a pedometer and brief verbal advice from an allied health care professional (not an exercise physiologist/exercise specialist) to monitor step counts and increase physical activity in line with guidelines, without providing any tailored or supervised training program, which enhances opportunities for clinical translation of the findings in the real world. A further strength is that there was >98% participant adherence to wearing a validated physical activity–monitoring device during the trial. This excellent adherence occurred despite the most common complaint from participants in the trial being discomfort caused by the accelerometer (e.g., rashes, which were managed by instructing participants to use the accelerometer on alternate arms on alternate days). Possible reasons for the high adherence with accelerometer wear could be that the accelerometer was given in person to each participant by 1 of the 2 dietitians working on the trial [except for 8 occasions out of all 606 scheduled occasions (1.3%), when the accelerometer was posted to the participant], along with verbal reminders on when and how to wear the accelerometer, and how to simultaneously use the food, activity, and sleep diary, which required participants to write down if and why they took the accelerometer off. These factors, combined with the high retention in the trial (discussed in what follows), may have contributed to the excellent adherence with accelerometer use.

The strengths of this study are associated with some weaknesses. One weakness is that the trial included only postmenopausal female adults with obesity, therefore the findings may not be generalizable beyond this population group. Another weakness is that we did not include a no-dietary restriction control group, so our study does not offer information on the efficacy of our low-resource physical activity intervention per se. However, a recent systematic review and meta-analysis found insufficient evidence that simple, self-monitored pedometer- or accelerometer-based interventions were associated with improvements in physical activity (36). A further weakness of this study is lack of blinding, as is the case for all dietary intervention studies where the diet that a participant has been randomly assigned to is apparent to the participant from what they are consuming. However, physical activity and sedentary time were objectively collected by the accelerometer, and participants could not see these data during or after the week in which the data were collected. An additional weakness is that all of the outcomes herein reported are secondary outcomes, differences in which the trial was not powered to detect. Hence, the conclusions presented in this article cannot be presented definitively.

The current findings were achieved within the context of a clinical trial involving 21–22 individual appointments with a trial dietitian in the first year, and the option of attending monthly group support sessions in years 2–3, along with efforts to build relations between trial participants and the same team over the 3 y. The trial had a 71.3% retention rate of participants at 36 mo (82.0% in the severe group, and 60.8% in the moderate group), which is high compared with some other weight loss trials of the same or shorter duration, where retention ranged from 97% at 18 mo to 15% at 2 mo, with typical retention being <75% at 12 mo (37). The intense intervention used in our trial likely contributed to this high retention, because a systematic review and meta-analysis showed that multicomponent interventions significantly increased retention in weight loss trials (37). Moreover, the older age (45–65 y) and generally highly educated status of participants in our trial may have also contributed to high retention, given that these factors appear to promote retention, as reported in a systematic review (38). It remains to be determined whether the same benefits of substantial weight loss on physical activity and sedentary time could be achieved using less intense support (e.g., less regular and/or peer support), in a different population of individuals. Another unanswered and related question is how to maintain the increases in physical activity and reductions in sedentary time beyond 12 mo. Because part of the effect of the interventions was achieved via the effect of the interventions to induce weight loss, it is likely that maintaining a low weight longer-term would promote longer-term effectiveness of the interventions on physical activity and sedentary time. Moreover, future research would benefit from investigating effects of substantial weight loss on the amount of muscle-strengthening exercise—an outcome not measured in this study—because muscle-strengthening exercise is also specifically recommended in the WHO 2020 Physical Activity Guidelines (30) and has been proposed as potentially important for preventing loss of bone mineral density during weight loss using a severely energy-restricted total meal replacement diet in postmenopausal female adults with obesity (32).

In conclusion, diet-induced loss of substantial body weight may be an effective strategy to increase physical activity and reduce sedentary time in postmenopausal female adults with obesity. Incorporating dietary weight loss interventions into clinical and public health strategies to promote physical activity and reduce sedentary time among individuals with obesity could enhance their effectiveness, particularly if the dietary weight loss intervention is a severely energy-restricted total meal replacement diet, implemented under clinical supervision, in part because these diets result in significantly greater proportions of people losing substantial amounts of body weight (21, 27, 32).

## Supplementary Material

nqac024_Supplemental_File

## Data Availability

Data are available on request by emailing the principal investigator of the TEMPO Diet Trial, AS (amanda.salis@uwa.edu.au).

## Notes

The TEMPO Diet Trial was supported by a National Health and Medical Research Council (NHMRC) of Australia project grant 1026005 (to AS and NMB). XJ, RVS, and SEK were supported by NHMRC Early Career Research Fellowships APP1143022, APP1072771, and APP122190, respectively. RVS was in addition supported by an Endocrine Society of Australia Postdoctoral Research Award. AAG was supported by an Australian Government Department of Education and Training Australian Postgraduate Award, then by an NHMRC Investigator Grant 1173784. ZS was the recipient of an Australian Government Department of Education, Skills and Employment Research Training Program Scholarship. ES was funded by NHMRC Senior Research Fellowship APP1110526 followed by NHMRC Investigator Grant Level 2 APP1194510. AS was supported by NHMRC Senior Research Fellowships APP1042555 and APP1135897. Both ES and AS were in addition supported by Sydney Outstanding Academic Researcher (SOAR) Fellowships from the University of Sydney. EI-E was supported by a fellowship from Poyraz Nakliyat, Samsun, Turkey. FQdL was supported by the CAPES Foundation, Ministry of Education of Brazil, via a postgraduate scholarship, and is now supported by São Paulo Research Foundation Young Investigator Grant 2018/18028-2 and Fellowship 2019/14622-0. Prima Health Solutions (Brookvale, New South Wales, Australia) provided in-kind support for this trial in the form of below-cost KicStart meal replacement products (shakes) and a gift of associated adherence tools (shakers). This relation with Prima Health Solutions was established after the dietary protocol for the TEMPO Diet Trial had been established.

The nongovernmental funders (Endocrine Society of Australia; The University of Sydney; Poyraz Nakliyat; São Paulo Research Foundation; and Prima Health Solutions) had no role in the design, implementation, analysis, or interpretation of the data.

Supplemental Tables 1 and 2 are available from the “Supplementary data” link in the online posting of the article and from the same link in the online table of contents at https://academic.oup.com/ajcn/.

XJ, AAG, and ZS contributed equally to this work as co–first authors.

Abbreviations used: MET, metabolic equivalent of task; MVPA, moderate-to-vigorous-intensity physical activity; Q-Q, quantile-quantile; SEREx, self-efficacy to regulate exercise; TEMPO, Type of Energy Manipulation for Promoting optimum metabolic health and body composition in Obesity.
