# Supplementary material for: Effect of severe compared with moderate energy restriction on physical activity among postmenopausal female adults with obesity: a prespecified secondary analysis of the Type of Energy Manipulation for Promoting optimum metabolic health and body composition in Obesity (TEMPO) Diet randomized controlled Trial
Source: Am J Clin Nutr. 2022 Jan 31;115(5):1393–403. doi: 10.1093/ajcn/nqac024 (PMC9071468; doi:10.1093/ajcn/nqac024)
Supplement: nqac024_Supplemental_File [file nqac024_supplemental_file.pdf]

Supplementary Table 1. Comparison of physical activity-related outcomes between the severe and moderate energy restriction interventions in the TEMPO Diet Trial

**Time point**	**Severe energy restriction**	**Moderate energy restriction**	**Between-group difference**	***P* value for the comparison between intervention groups at that month**
	**Estimated marginal mean [95% confidence interval]**			
**Total volume of physical activity, MET-minutes per week**<br>***P* value for the interaction between intervention group × time point < 0.0001**				
Month 0	3716<br>[3421, 4012]	3701<br>[3406, 3997]	15<br>[-403, 433]	0.9425
Month 0.25	3593<br>[3290, 3897]	3670<br>[3369, 3971]	-76<br>[-504, 351]	0.7251
Month 1	3854<br>[3550, 4157]	3749<br>[3448, 4050]	105<br>[-322, 532]	0.6290
Month 4	4818<br>[4507, 5129] $^{P < 0.0001}$	4008<br>[3680, 4335]	810<br>[358, 1262]	**0.0005**
Month 6	5087<br>[4786, 5388] $^{P < 0.0001}$	4080<br>[3756, 4405]	1006<br>[564, 1449]	**< 0.0001**
Month 12	4827<br>[4521, 5134] $^{P < 0.0001}$	4111<br>[3776, 4446]	717<br>[263, 1170]	**0.0020**
Month 24	4278<br>[3963, 4592] $^{P = 0.0025}$	4341<br>[3981, 4702] $^{P = 0.0020}$	-64<br>[-542, 414]	0.7934
Month 36	4279<br>[3952, 4605] $^{P = 0.0033}$	3849<br>[3461, 4238]	429<br>[-78, 937]	0.0971
**Moderate-to-vigorous-intensity physical activity, minutes per week**<br>***P* value for the interaction between intervention group × time point = 0.0001**				
Month 0	326<br>[275, 377]	328<br>[277, 378]	1<br>[-73, 70]	0.9686
Month 0.25	318<br>[266, 370]	335<br>[283, 386]	-16<br>[-90, 57]	0.6603
Month 1	347<br>[295, 399]	365<br>[313, 417]	-18<br>[-91, 55]	0.6281
Month 4	465<br>[412, 519] $^{P < 0.0001}$	369<br>[312, 425]	96<br>[19, 174]	0.0150
Month 6	502<br>[450, 553] $^{P = 0.0001}$	354<br>[299, 410]	147<br>[71, 223]	**0.0002**
Month 12	505<br>[453, 558] $^{P = 0.0001}$	381<br>[323, 438]	124<br>[47, 202]	**0.0018**
Month 24	452<br>[398, 506] $^{P = 0.0001}$	453<br>[391, 515] $^{P = 0.0004}$	-1<br>[-83, 81]	0.9773
Month 36	441<br>[385, 497] $^{P = 0.0005}$	406<br>[339, 472]	35<br>[-52, 122]	0.4294

Supplementary Table 1 ***continued.*** Comparison of physical activity-related outcomes between the severe and moderate energy restriction interventions in the TEMPO Diet Trial

Time point	Severe energy restriction	Moderate energy restriction	Between-group difference	*P* value for the comparison between intervention groups at that month
**Light-intensity physical activity, minutes per week** ***P* value for the interaction between intervention group × time point = 0.0194**				
Month 0	1323 [1230, 1415]	1307 [1214, 1400]	16 [-116, 147]	0.8131
Month 0.25	1279 [1183, 1374]	1281 [1186, 1375]	-2 [-137, 132]	0.9733
Month 1	1357 [1262, 1453]	1262 [1167, 1356]	95 [-39, 230]	0.1636
Month 4	1618 [1520, 1716] $^{P < 0.0001}$	1402 [1299, 1505]	216 [74, 358]	**0.0029**
Month 6	1683 [1588, 1777] $^{P < 0.0001}$	1472 [1370, 1575] $^{P = 0.0065}$	210 [71, 350]	**0.0031**
Month 12	1554 [1457, 1650] $^{P = 0.0001}$	1431 [1326, 1537]	122 [-20, 265]	0.0929
Month 24	1368 [1269, 1467]	1411 [1297, 1524]	-42 [-193, 108]	0.5826
Month 36	1388 [1285, 1491]	1261 [1139, 1384]	126 [-34, 286]	0.1219
**Steps, count per day** ***P* value for the interaction between intervention group × time point = 0.0123**				
Month 0	7214 [6704, 7724]	7211 [6701, 7721]	3 [-718, 724]	0.9934
Month 0.25	7190 [6666, 7713]	7604 [7085, 8124]	-415 [-1152, 323]	0.2698
Month 1	7374 [6850, 7898]	7899 [7380, 8419]	-526 [-1263, 212]	0.1620
Month 4	7754 [7216, 8292]	7546 [6980, 8112]	208 [-573, 989]	0.6009
Month 6	8206 [7686, 8725] $^{P = 0.0016}$	7290 [6730, 7851]	915 [151, 1680]	0.0190
Month 12	7782 [7254, 8311]	7159 [6580, 7738]	623 [-161, 1407]	0.1191
Month 24	8090 [7546, 8633] $^{P = 0.0068}$	7541 [6918, 8165]	548 [-279, 1375]	0.1936
Month 36	7667 [7102, 8232]	6964 [6292, 7636]	703 [-175, 1581]	0.1163

Supplementary Table 1 *continued.* Comparison of physical activity-related outcomes between the severe and moderate energy restriction interventions in the TEMPO Diet Trial

**Time point**	**Severe energy restriction**	**Moderate energy restriction**	**Between-group difference**	***P* value for the comparison between intervention groups at that month**
**Sedentary time, minutes per day**<br>***P* value for the interaction between intervention group × time point = 0.0179**				
Month 0	1168<br>[1146, 1189]	1170<br>[1149, 1192]	-3<br>[-33, 28]	0.8622
Month 0.25	1163<br>[1140, 1185]	1158<br>[1136, 1179]	5<br>[-26, 36]	0.7555
Month 1	1137<br>[1115, 1159]	1152<br>[1130, 1174]	-15<br>[-46, 16]	0.3563
Month 4	1090<br>[1068, 1113] $^{P<0.0001}$	1123<br>[1099, 1147] $^{P=0.0014}$	-32<br>[-66, 1]	0.0548
Month 6	1063<br>[1041, 1085] $^{P<0.0001}$	1109<br>[1085, 1133] $^{P<0.0001}$	-46<br>[-78, -14]	**0.0054**
Month 12	1078<br>[1056, 1101] $^{P<0.0001}$	1115<br>[1090, 1139] $^{P=0.0002}$	-36<br>[-70, -3]	0.0317
Month 24	1141<br>[1118, 1164]	1124<br>[1097, 1150] $^{P=0.0035}$	17<br>[-18, 52]	0.3435
Month 36	1117<br>[1093, 1141] $^{P=0.0006}$	1160<br>[1132, 1189]	-44<br>[-81, -6]	0.0228
**Self-Efficacy to Regulate Exercise score, 0 to 100**<br>***P* value for the interaction between intervention group × time point** = 0.6382				
Month 0	47.2<br>[43.0, 51.3]	43.7<br>[39.7, 47.8]		
Month 0.25	Not determined at this time point			
Month 1	49.0<br>[44.8, 53.1]	45.5<br>[41.4, 49.7]		
Month 4	51.2<br>[47.0, 55.4]	47.8<br>[43.6, 52.0]		
Month 6	52.4<br>[48.2, 56.5]	48.9<br>[44.7, 53.1]		
Month 12	51.2<br>[46.9, 55.4]	47.7<br>[43.5, 52.0]		
Month 24	50.7<br>[46.3, 55.0]	47.2<br>[42.8, 51.7]		
Month 36	49.4<br>[44.9, 53.8]	46.0<br>[41.4, 50.5]		

Analyses were performed on an intention-to-treat basis (n = 101: 50 in the severe intervention group; 51 in the moderate intervention group). Data are the estimated marginal means (i.e., group means after controlling for covariates) and their 95% confidence intervals from a repeated measures linear mixed-effect model. Missing data were handled by the restricted maximum likelihood estimation function in the linear mixed-effect model. *P* values lower than 0.0071, which is our Bonferroni-adjusted threshold for statistical significance (i.e., 0.05 divided by the number of comparisons being made, which was 7 because there were 7 time points), are defined as being statistically significant and are shown in bold text. The *P* values shown in superscript text are for the pair-wise comparison from baseline (month 0) within that intervention group to that time point, with *P* values only being shown if they are below our Bonferroni-adjusted threshold of 0.0071. In empty cells, the between-group difference (and corresponding *P* value for the comparison between intervention groups at that month) are not relevant because the *P* value for the interaction between intervention group × time point was not less than 0.05.

Supplementary Table 2. Comparison of physical activity-related outcomes between the severe and moderate energy restriction interventions in the TEMPO Diet Trial, adjusted for weight

**Time point**	**Severe energy restriction**	**Moderate energy restriction**	**Between-group difference**	***P* value for the comparison between intervention groups at that month**
	**Estimated marginal mean [95% confidence interval]**			
**Total volume of physical activity, MET-minutes per week**<br>***P* value for the interaction between intervention group × time point = 0.0130**				
Month 0	3955<br>[3663, 4248]	4016<br>[3716, 4316]	-60<br>[-460, 340]	0.7669
Month 0.25	3725<br>[3432, 4019]	3934<br>[3634, 4234]	-209<br>[-620, 202]	0.3182
Month 1	3848<br>[3558, 4138]	3916<br>[3618, 4214]	-68<br>[-484, 348]	0.7481
Month 4	4407<br>[4080, 4733]	4060<br>[3745, 4375]	347<br>[-112, 806]	0.1377
Month 6	4659<br>[4339, 4978] $^{P = 0.0007}$	4076<br>[3765, 4387]	582<br>[137, 1028]	0.0106
Month 12	4488<br>[4176, 4801]	4101<br>[3780, 4423]	387<br>[-61, 835]	0.0903
Month 24	4147<br>[3843, 4451]	4409<br>[4061, 4757]	-262<br>[-727, 202]	0.2671
Month 36	4211<br>[3897, 4525]	3956<br>[3579, 4332]	256<br>[-236, 748]	0.3078
**Moderate-to-vigorous-intensity physical activity, minutes per week**<br>***P* value for the interaction between intervention group × time point = 0.0116**				
Month 0	353<br>[301, 404]	364<br>[311, 418]	-12<br>[-82, 59]	0.7491
Month 0.25	333<br>[281, 385]	365<br>[312, 419]	-33<br>[-106, 40]	0.3762
Month 1	346<br>[294, 397]	385<br>[332, 438]	-39<br>[-113, 34]	0.2951
Month 4	418<br>[360, 476]	375<br>[319, 431]	43<br>[-38, 124]	0.3012
Month 6	452<br>[395, 509] $^{P = 0.0064}$	354<br>[299, 409]	98<br>[19, 38]	0.0155
Month 12	466<br>[410, 522] $^{P = 0.0014}$	380<br>[323, 437]	86<br>[7, 166]	0.0337
Month 24	437<br>[383, 491]	461<br>[400, 523] $^{P = 0.0069}$	-25<br>[-107, 57]	0.5551
Month 36	433<br>[377, 488]	418<br>[352, 485]	15<br>[-72, 101]	0.7409

Supplementary Table 2 *continued.* Comparison of physical activity-related outcomes between the severe and moderate energy restriction interventions in the TEMPO Diet Trial, adjusted for weight

**Time point**	**Severe energy restriction**	**Moderate energy restriction**	**Between-group difference**	***P* value for the comparison between intervention groups at that month**
**Light-intensity physical activity, minutes per week**<br>***P* value for the interaction between intervention group × time point** = 0.3766				
Month 0	1410<br>[1336, 1485]	1396<br>[1314, 1479]		
Month 0.25	1350<br>[1275, 1425]	1336<br>[1256, 1417]		
Month 1	1343<br>[1269, 1418]	1330<br>[1252, 1407]		
Month 4	1462<br>[1380, 1544]	1448<br>[1370, 1527]		
Month 6	1519<br>[1437, 1601]	1505<br>[1428, 1583]		
Month 12	1445<br>[1363, 1527]	1431<br>[1352, 1510]		
Month 24	1377<br>[1296, 1458]	1363<br>[1280, 1446]		
Month 36	1344<br>[1260, 1427]	1330<br>[1243, 1417]		
**Steps, count per day**<br>***P* value for the interaction between intervention group × time point = 0.0468**				
Month 0	7445<br>[6922, 7969]	7524<br>[6985, 8062]	-78<br>[-794, 637]	0.8296
Month 0.25	7316<br>[6792, 7841]	7868<br>[7330, 8406]	-552<br>[-1286, 183]	0.1409
Month 1	7365<br>[6847, 7884]	8082<br>[7549, 8616]	-717<br>[-1462, 28]	0.0591
Month 4	7347<br>[6761, 7933]	7604<br>[7042, 8166]	-257<br>[-1080, 566]	0.5395
Month 6	7783<br>[7209, 8356]	7292<br>[6737, 7848]	490<br>[-1290, 309]	0.5395
Month 12	7446<br>[6885, 8007]	7157<br>[6583, 7730]	-289<br>[-513, 1092]	0.4791
Month 24	7958<br>[7414, 8501]	7613<br>[6993, 8233]	345<br>[-484, 1174]	0.4140
Month 36	7600<br>[7039, 8161]	7073<br>[6403, 7744]	526<br>[-351, 1403]	0.2391

Supplementary Table 2 *continued.* Comparison of physical activity-related outcomes between the severe and moderate energy restriction interventions in the TEMPO Diet Trial, adjusted for weight

**Time point**	**Severe energy restriction**	**Moderate energy restriction**	**Between-group difference**	***P* value for the comparison between intervention groups at that month**
**Sedentary time, minutes per day**<br>***P* value for the interaction between intervention group × time point** = 0.0920				
Month 0	1153<br>[1135, 1170]	1161<br>[1141, 1180]		
Month 0.25	1148<br>[1130, 1165]	1155<br>[1136, 1174]		
Month 1	1137<br>[1119, 1154]	1144<br>[1126, 1163]		
Month 4	1110<br>[1091, 1130]	1118<br>[1099, 1137]		
Month 6	1090<br>[1071, 1110]	1098<br>[1080, 1117]		
Month 12	1099<br>[1080, 1119]	1107<br>[1088, 1126]		
Month 24	1132<br>[1113, 1151]	1140<br>[1120, 1159]		
Month 36	1131<br>[1111, 1151]	1139<br>[1118, 1159]		
**Self-Efficacy to Regulate Exercise score, 0 to 100**<br>***P* value for the interaction between intervention group × time point** = 0.6037				
Month 0	48.9<br>[44.7, 53.1]	48.1<br>[43.5, 52.7]		
Month 0.25	Not determined at this time point			
Month 1	49<br>[44.8, 53.1]	48.2<br>[43.9, 52.6]		
Month 4	48.4<br>[43.9, 52.8]	47.6<br>[43.4, 51.8]		
Month 6	49.1<br>[44.6, 53.6]	48.4<br>[44.2, 52.6]		
Month 12	48.4<br>[44, 52.9]	47.7<br>[43.4, 51.9]		
Month 24	49.4<br>[45, 53.8]	48.6<br>[44.1, 53.1]		
Month 36	48.7<br>[44.2, 53.1]	47.9<br>[43.3, 52.5]		

Analyses were performed on an intention-to-treat basis (n = 101: 50 in the severe intervention group; 51 in the moderate intervention group). Data are the estimated marginal means (i.e., group means after controlling for covariates) and their 95% confidence intervals from a repeated measures linear mixed-effect model that included weight at each time point as a covariate. Missing data were handled by the restricted maximum likelihood estimation function in the linear mixed-effect model. *P* values lower than 0.0071, which is our Bonferroni-adjusted threshold for statistical significance (i.e., 0.05 divided by the number of comparisons being made, which was 7 because there were 7 time points), are defined as being statistically significant and are shown in bold text. The *P* values shown in superscript text are for the pair-wise comparison from baseline (month 0) within that intervention group to that time point, with *P* values only being shown if they are below our Bonferroni-adjusted threshold of 0.0071. In empty cells, the between-group difference (and corresponding *P* value for the comparison between intervention groups at that month) are not relevant because the *P* value for the interaction between intervention group × time point was not less than 0.
